# *Mycobacterium tuberculosis* lineage 4 associated with cavitations and treatment failure

**DOI:** 10.1186/s12879-023-08055-9

**Published:** 2023-03-14

**Authors:** Anabel Ordaz-Vázquez, Pedro Torres-González, Leticia Ferreyra-Reyes, Sergio Canizales-Quintero, Guadalupe Delgado-Sánchez, Lourdes García-García, Alfredo Ponce-De-León, José Sifuentes-Osornio, Miriam Bobadilla-Del-Valle

**Affiliations:** 1 Departamento de Infectología, Instituto Nacional de Ciencias Médicas y Nutrición Salvador Zubirán, Vasco de Quiroga 15, Belisario Domínguez Sección XVI, Tlalpan, 14080 Mexico City, Mexico; 2 Centro de Investigación en Enfermedades Infecciosas, Instituto Nacional de Salud Pública, Cuernavaca, Mexico

**Keywords:** *Mycobacterium tuberculosis*, Lineage 4, Treatment failure, Cavitations, Clinical association

## Abstract

**Background:**

*Mycobacterium tuberculosis* genotyping has been crucial to determining the distribution and impact of different families on disease clinical presentation. The aim of the study was to evaluate the associations among sociodemographic and clinical characteristics and *M. tuberculosis* lineages from patients with pulmonary tuberculosis in Orizaba, Veracruz, Mexico.

**Methods:**

We analyzed data from 755 patients whose isolates were typified by 24-*loci* mycobacterial interspersed repetitive unit–variable number of tandem repeats (MIRU–VNTR). The associations among patient characteristics and sublineages found were evaluated using logistic regression analysis.

**Results:**

Among *M. tuberculosis* isolates, 730/755 (96.6%) were assigned to eight sublineages of lineage 4 (Euro-American). Alcohol consumption (adjusted odds ratio [aOR] 1.528, 95% confidence interval (CI) 1.041–2.243; p = 0.030), diabetes mellitus type 2 (aOR 1.625, 95% CI 1.130–2.337; p = 0.009), sputum smear positivity grade (3+) (aOR 2.198, 95% CI 1.524–3.168; p < 0.001) and LAM sublineage isolates (aOR 1.023, 95% CI 1.023–2.333; p = 0.039) were associated with the presence of cavitations. Resistance to at least one drug (aOR 25.763, 95% CI 7.096–93.543; p < 0.001) and having isolates other than Haarlem and LAM sublineages (aOR 6.740, 95% CI 1.704–26.661; p = 0.007) were associated with treatment failure. In a second model, multidrug resistance was associated with treatment failure (aOR 31.497, 95% CI 5.119–193.815; p < 0.001). Having more than 6 years of formal education was not associated with treatment failure.

**Conclusions:**

Knowing *M. tuberculosis* genetic diversity plays an essential role in disease development and outcomes, and could have important implications for guiding treatment and improving tuberculosis control.

**Supplementary Information:**

The online version contains supplementary material available at 10.1186/s12879-023-08055-9.

## Background

The agent responsible for tuberculosis belongs to *Mycobacterium tuberculosis* complex (MTBC). Pulmonary tuberculosis is the most common disease presentation, reported in 4.8 million cases worldwide [1]. The state of Veracruz in southern Mexico reports the highest number of cases (2198) nationwide [2]. The incidence in the municipality of Orizaba, Veracruz was 16–38 cases/100,000 inhabitants during the period 1995–2010 surpassing the national incidence [3].

Nowadays, nine *M. tuberculosis* lineages have been identified strongly associated with particular geographic regions [4, 5]. In the Americas, tuberculosis is mainly caused by lineage 4 also known as Euro-American lineage [6].

*Mycobacterium tuberculosis* genotyping is important because it contributes to knowledge regarding its genetic diversity [7, 8]. The current gold standard for genotyping is mycobacterial interspersed repetitive unit–variable number of tandem repeat (MIRU–VNTR) method. Currently, MIRUs are used as markers for strains classification and sub-classification. For example, within the Latin American & Mediterranean (LAM) family, a single repeat of MIRU40 has been proposed as a marker of RD^Rio^ sub-lineage [9].

Risk factors related to *M. tuberculosis* genetics help in the early identification of patients infected with lineages associated with increased risk of treatment failure, relapse, drug resistance and death [10]. External risk factors associated with active tuberculosis development are poverty, overpopulation, overcrowding and malnutrition, in addition to comorbidities such as human immunodeficiency virus (HIV) coinfection, diabetes mellitus type 2 (DM2), chronic kidney failure, silicosis, immunosuppressive therapies and addictions such as smoking and drinking [11, 12].

In addition to host and environmental risk factors, tuberculosis epidemiology can also be influenced by *M. tuberculosis* genetic diversity [13]*.* Some lineages have shown differences in their virulence phenotypes, affecting transmissibility and pathogenesis and having implications in treatment outcomes and failure in the effectiveness of the BCG vaccine [6, 14].

The aim of this study was to evaluate the association among sociodemographic and clinical characteristics and *M. tuberculosis* lineages from isolates of patients with pulmonary tuberculosis obtained in a population-based study conducted in Orizaba, Veracruz, Mexico from 1995 to 2010.

## Methods

### Study population and data collection

Between March 1995 and April 2010, pulmonary tuberculosis cases passive search was carried out in people over 15 years of age who had respiratory symptoms for more than two weeks in 12 health jurisdictions municipality of Orizaba, Veracruz, Mexico. During this period, 1132 patients were diagnosed and for this study 612 M*. tuberculosis* isolates were recovered from a strain collection and 143 more from a DNA collection using samples from these patients. We used the population-based cohort data from patients diagnosed with pulmonary tuberculosis from August 1, 1997, to April 30, 2010. The study was approved by the Ethics Committee (Ref. No. 1515). All participating patients signed informed consent forms.

As part of the cohort investigation, isolates were genotyped by 24-*loci* MIRU–VNTR and susceptibility tests were performed as previously described [15]. LAM RD^Rio^ and RD115 sublineages were classified according to the presence of a single repeat in MIRU40 and MIRU02 respectively.

### Definitions

The following sociodemographic variables were considered: sex, age, education level, dirt-floor home, and rural residence locality, nearest health center distance, social security access, and consumption of alcohol, tobacco and illicit drugs. DM2 and HIV diagnosis was also considered. Presence of acid-fast bacilli (AFB) in sputum samples information was considered and was graded as follows: 1 + (1–9 bacilli per 100 observed fields), 2 + (1–9 bacilli per 10 observed fields) or 3 + (1–9 bacilli per observed field). We included fever, hemoptysis and presence of cavitations variables, each used dichotomously. Body mass index (BMI) and number of days between symptom onset and start of treatment were calculated.

We used tuberculosis prevention and control program (NOM-SSA-006) operational definitions for treatment outcomes, except failure and death, which were defined according to international definitions [16, 17]: *cure*, patient who completed treatment, with signs and symptoms disappearance, or patient who had smear or culture negative at the end of treatment; *failure*, patient who had smear or culture positive after five months or later during treatment; and *treatment completion*, patient who completed his/her treatment regimen with signs and symptoms disappearance and smear or culture were not performed. Patients who did not complete treatment were classified into the following two categories: *abandon*, patient who interrupts treatment for 30 days or more; and *death during treatment*, patient who died due to any other cause during treatment.

Lineage variable was operationalized in disaggregated and aggregate way according to MIRU–VNTR genotyping. Disaggregated variable considers each identified sublineage, Haarlem, LAM, Cameroon, UgandaI, Ghana, S, X, TUR, EAI, Beijing and unknown. Aggregate variable considers lineage frequency, Haarlem, LAM and lineages other than Haarlem and LAM, because of the small frequency of each other lineages.

### Statistical analysis

We calculated the distributions percentage for qualitative variables as well as medians and interquartile ranges (IQR) for quantitative variables. We used Pearson chi-square test for dichotomous variables, binomial test for categorical variables and Kruskal–Wallis test for quantitative variables. Unconditional logistic regression models were adjusted to explain treatment failure and the presence of cavitation on radiography. Two models were adjusted to explain treatment failure: one included resistance variable to at least one drug, and the other included MDR. To include variables in a multivariate model were considered those that in the bivariate analysis had values of *p* ≤ 0.20 and biological plausibility. We estimated adjusted odds ratio (aOR) and 95% confidence intervals (CIs).

Analyses were performed using STATA^®^ v15 statistical software package (StataCorp LP, College Station, TX, USA).

## Results

The characteristics of the studied patients are shown in Table 1. The proportion of individuals among the population examined was similar to the proportion represented by this same group. A total of 755 patients were included in the study, 442 (59%) of whom were men, with a median age of 45 years (IQR 32–59). There were 507 (67%) patients with more than six years of formal education, and 174 (23%) lived in dirt-floor homes. Comorbidity with DM2 was reported in 250 (33%) patients. HIV status was known for 739 patients, of whom 13 (2%) were positive. Resistance to any drug was present in 116/612 (19%) isolates, and 20 (3%) were MDR. The most common clinical findings were fever and cavitation in 531/752 (71%) and 282/626 (45%) patients respectively. Cure was recorded in 532/755 (70%) patients.

**Table 1:** Demographic and clinical characteristics of the patients with pulmonary tuberculosis in Orizaba, Veracruz, 1995–2010 (n = 1132)

Variables	Total cases	Sample percentage
	n/total^a^ (%)	n/total^a^ (%)
Male	654/1132 (58.0)	442/755 (59.0)
Age (years) (median [IQR])	47 [32–60]	45 [32–59]
> 6 years of formal schooling	789/1131 (70.0)	507/754 (67.0)
House with dirt floor	218/1132 (19.0)	174/755 (23.0)
Rural residence	134/1002 (13.0)	99/732 (14.0)
Nearest health center distance (meters) (median [IQR])	698 [412–1073]	708 [414–1099]
Access to social security	400/1132 (35.0)	254/755 (34.0)
Used alcohol	468/1130 (41.0)	330/753 (44.0)
Used tobacco	222/1129 (20.0)	164/753 (22.0)
Used illegal drug	50/1131 (4.0)	35/754 (5.0)
Homelessness or residence in shelters	33/1129 (3.0)	20/754 (3.0)
Diabetes mellitus type 2	386/1132 (34.0)	250/755 (33.0)
HIV coinfection	19/1095 (2.0)	13/739 (2.0)
New case	934/1131 (83.0)	688/754 (91.0)
AFB sputum positivity grade of 3+	281/1132 (24.8)	211/755 (28.0)
Resistance to at least one drug	176/826 (21.0)	116/612 (19.0)
MDR	47/826 (6.0)	20/612 (3.0)
Fever	744/1129 (66.0)	531/752 (71.0)
Haemoptysis	352/1126 (31.0)	250/753 (33.0)
Cavitations presence on chest radiograph	398/927 (43.0)	282/626 (45.0)
BMI (median [IQR])	21.2 [18.6–24.0]	20.9 [18.4–23.8]
Number of days between symptom onset and start of treatment (median [IQR])	104 [63–186]	105 [67–182]
*Treatment outcome*		
Abandon	86/1132 (8.0)	49/755 (6.0)
Cure	756/1132 (67.0)	532/755 (70.0)
Treatment completion	136/1132 (12.0)	93/755 (12.0)
Failure	26/1132 (2.0)	20/755 (3.0)
Death during treatment	49/1132 (4.0)	26/755 (3.0)
No data	79/1132 (7.0)	35/755 (5.0)
*Treatment failure*		
Failure	26/918 (2.8)	20/645 (3.1)
Cure or treatment completion	892/918 (97.2)	625/645 (96.9)
*No treatment success*		
Abandon, failure or death during treatment	161/1053 (15.3)	95/720 (13.2)
Cure or treatment completion	892/1053 (84.7)	625/720 (86.8)
*Death during treatment*		
Death	49/1053 (4.7)	26/720 (3.6)
Cure or treatment completion, abandon or failure	1004/1053 (95.4)	794/720 (96.4)

IQR, interquartile range; HIV, human immunodeficiency virus; AFB, acid fast bacilli; MDR, multidrug resistance; BMI, body mass index

^a^Because there were missing values for the characteristics of some of the tuberculosis patients, several of the numbers below do not sum to the group total

**Fig. 1:**
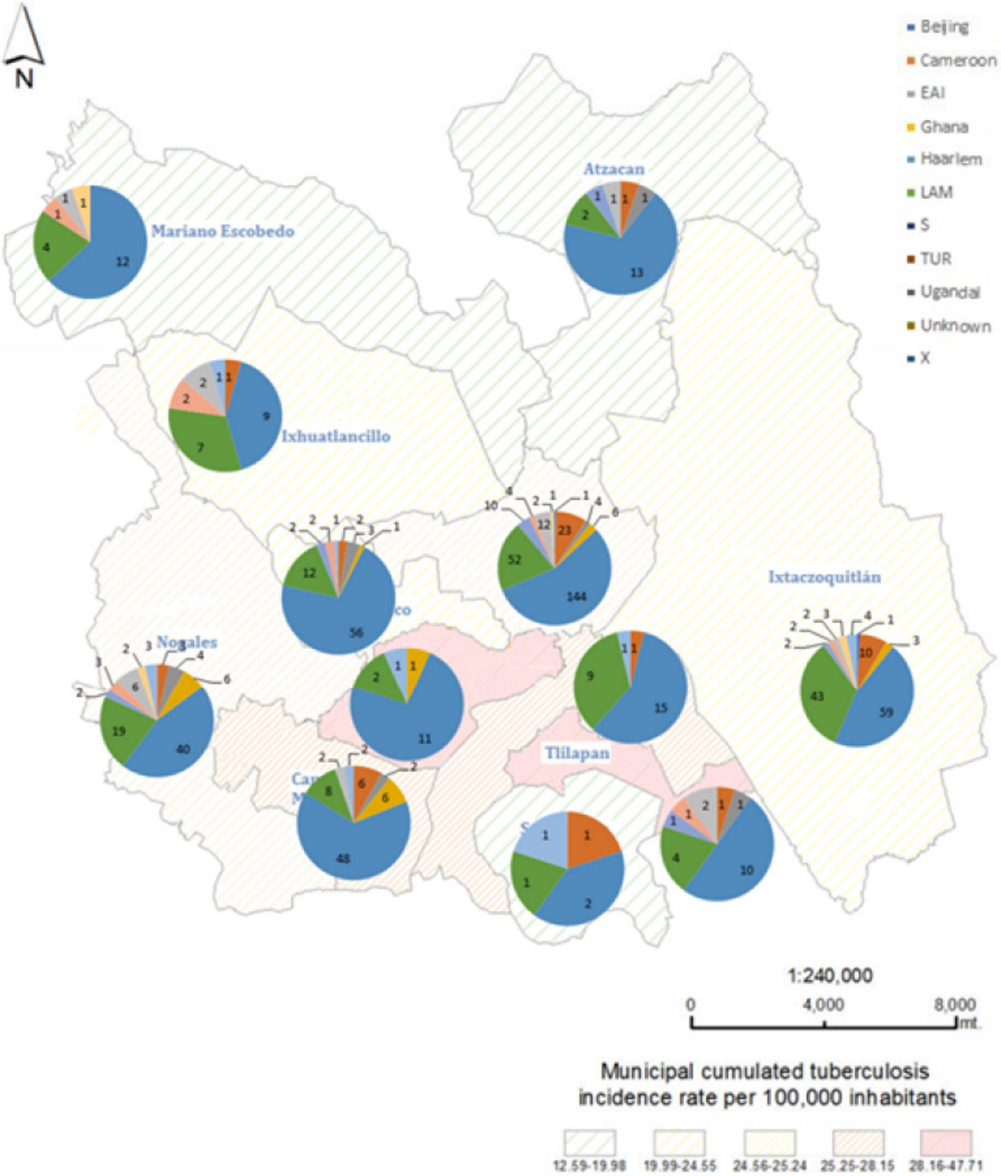
Map of the distribution of *M. tuberculosis* isolates in the jurisdiction of Orizaba, Mexico

We identified ten sublineages among the 755 *M. tuberculosis* isolates (one from each patient) genotyped by 24-*loci* MIRU–VNTR. The most frequent sublineages were Haarlem (419, 55.5%) and LAM (163, 21.6%) which was sub classified in RD^Rio^ (114/163, 69.9%) characterized by one repeat in MIRU40 and RD115 (31/163, 19%) characterized by one repeat in MIRU02, followed by Cameroon (49, 6.5%), Uganda I (28, 3.7%), Ghana (23, 3%), S (18, 2.4%), X (15, 2%), TUR (15, 2%), EAI (15, 2%) and Beijing (2, 0.2%). It was not possible to determine lineage in eight isolates (1.1%) and therefore, these were considered unknown, the distribution of *M. tuberculosis* isolates in the jurisdiction of Orizaba, Veracruz is shown in Fig. 1.

Data analysis revealed that Haarlem sublineage had the highest proportion (318/419, 75.9%) of clustered patients compared with other sublineages. Patients with Cameroon sublineage isolates had more days between symptom onset and start of treatment (median 129, IQR 83–198), and patients isolates with Ghana sublineage presented hemoptysis (11/23, 47.8%) with greater frequency compared with the other sublineages. EAI sublineage isolates were more frequent in men (12/15, 80.0%), in DM2 patients (10/15, 66.7%) and in patients who had ever smoked (7/15, 46.7%). Patients with Beijing sublineage isolates were older (median 59 years, IQR 57–60) than patients with other sublineages; one of the two patients had HIV (1/2, 50%), the other had DM2 besides the isolate was MDR (1/2, 50%), both showed higher BMI (median 25.4, IQR 21.6–29.3) compared to patients with other sublineages (Additional file 1: Table S1).

The possible association between sublineages and population clinical characteristics was explored, grouping patients with Haarlem, LAM and lineages other than Haarlem and LAM (Table 2). Significantly, most of the patients with Haarlem lineage were men (61.3%, 247/419); 25.4% (106/417) had ever smoked, had a median BMI of 20.5 (IQR 18.1–23.4), lower than the average, and 75.9% (318/419) were found clustered (*p* < 0.05). Significantly more patients with LAM lineage presented cavitation 54.5% (74/136) as radiographic finding (*p* = 0.023). In patients with sublineages other than Haarlem and LAM 40.5% (70/173) had hemoptysis as the most common clinical feature (*p* = 0.005).

**Table 2:** Sociodemographic and clinical characteristics of patients with pulmonary tuberculosis according to sublineage aggregated, determined by 24-*loci* MIRU–VNTR, Orizaba Veracruz 1997–2010 (n = 755)

Variables	Total	Haarlem	LAM	Other than Haarlem and LAM	*p-*value*
	n/total (%)	n/total (%)	n/total (%)	n/total (%)	
Male	442/755 (58.5)	257/419 (61.3)	81/163 (49.7)	104/173 (60.1)	0.034
Age (years) (median [IQR])	45 (32–59)	45 (33–59)	43 (30–55)	47 (31–60)	0.503†
> 6 years of formal schooling	507/754 (67.2)	277/418 (66.3)	115/163 (70.6)	115/173 (24.3)	0.595
House with dirt floor	174/755 (23.1)	88/419 (21.0)	44/163 (27.0)	42/173 (24.0)	0.277
Rural residence	99/732 (13.5)	47/409 (11.5)	30/159 (18.9)	22/164 (13.4)	0.070
Nearest health center distance (meters) (median [IQR])	708 (414–1099)	775 (432–1184)	775 (432–1183.7)	737 (455–1118)	0.241†
Access to social security	254/755 (33.6)	152/419 (36.3)	48/163 (29.5)	54/173 (31.2)	0.218
Used alcohol	330/753 (43.8)	194/417 (46.5)	64/163 (39.3)	72/173 (41.6)	0.229
Used tobacco	164/753 (21.8)	106/417 (25.4)	22/163 (13.5)	36/173 (20.8)	0.007
Used illegal drugs	35/754 (4.6)	19/418 (4.6)	5/163 (3.1)	11/173 (6.4)	0.355
Homelessness or residence in shelters	20/754 (2.7)	12/419 (2.9)	4/162 (2.5)	4/173 (2.3)	0.918
Diabetes mellitus type 2	250/755 (33.1)	138/419 (32.9)	52/163 (31.9)	60/173 (34.7)	0.858
HIV coinfection	13/739 (1.8)	7/410 (1.7)	3/163 (1.8)	3/166 (1.8)	0.993
New case	688/754 (91.3)	384/418 (91.9)	145/163 (89.0)	159/173 (91.9)	0.505
AFB sputum positivity grade of 3+	211/755 (28.0)	123/419 (29.4)	47/163 (28.8)	41/173 (23.7)	0.363
Resistance to at least one drug	117/612 (19.1)	68/337 (20.2)	25/131 (19.1)	24/144 (16.7)	0.669
MDR	21/612 (3.4)	8/337 (2.4)	8/131 (6.1)	5/144 (3.5)	0.138
Fever	531/752 (70.6)	292/417 (70.0)	114/162 (70.4)	125/173 (72.3)	0.861
Haemoptysis	250/753 (33.2)	141/417 (33.8)	39/163 (23.9)	70/173 (40.5)	0.005
Cavitations presence on chest radiograph	282/626 (45.1)	152/344 (44.2)	74/136 (54.5)	56/146 (38.4)	0.023
BMI (median [IQR])	20.9(18.4–23.8)	20.5(18.1–23.4)	21.0(18.9–24.1)	21.6(19.0–23.9)	0.016†
Number of days between symptom onset and start of treatment (median [IQR])	105 (67–182)	106 (63–180)	119 (80–196)	97 (66.5–175)	0.098†
Cluster belonging	519/755 (68.7)	318/419 (75.9)	95/163 (58.3)	106/173 (61.3)	< 0.001

IQR, interquartile range; HIV, human immunodeficiency virus; AFB, acid fast bacilli; MDR, multidrug resistance; BMI, body mass index

*Pearson chi-square test

^†^Kruskal–Wallis test

Regarding treatment outcomes, we compared cure or treatment completion with treatment failure in all identified lineages. It was observed that patients with X sublineage isolates (2/11, 18.2%) and unknown isolates (1/7, 14.3%) had the highest failure rates, followed by Cameroon (3/41, 7.3%), EAI (1/15, 6.7%), S (1/18, 5.6%), LAM (6/135, 4.4%), Uganda I (1/26, 3.9%) and Haarlem (5/358, 1.4%). Patients with Beijing, Ghana and TUR lineages did not experience treatment failure (Additional file 1: Table S2).

Treatment outcome according to sublineage is summarized in Additional file 1: Table S3. When comparing cure or completion with treatment failure, patients with sublineages other than Haarlem and LAM showed higher proportion of treatment failure (9/152, 5.9%) than patients with Haarlem (1.4%) and LAM (4.4%) lineages (p = 0.016).

The comparison among clinical characteristics according to cure or treatment completion compared to failure, revealed higher proportion of treatment failure in patients who had ever smoked (8/20, 40% vs 131/624, 21.0%; p = 0.034), showed resistance to at least one drug (13/17, 76.0% vs 80/505, 16.0%; p < 0.001), showed MDR presence (5/17, 2.0% vs 6/505, 1.0%, p < 0.001) and had sublineages other than Haarlem and LAM (9/20, 45.0% vs 143/625, 23.0%; p = 0.022) (Table 3). We observed higher proportion of cure in patients who had formal education > 6 years (423/625, 68.0% vs 9/20, 45%; p = 0.034), had Haarlem sublineage (353/625, 56.5% vs 5/20, 25%; p = 0.005) and presented LAM RD^Rio^ sublineage (1/6, 16.7% vs 92/129, 71.3%; p = 0.005).

**Table 3:** Sociodemographic and clinical characteristics of patients with pulmonary tuberculosis by treatment outcome, Orizaba Veracruz 1997–2010 (n = 755)

Variables	Total	Failure	Cure or treatment completion	*p*-value*
	n/total^a^ (%)	n/total (%)	n/total (%)	
Male	363/645 (56.0)	15/20 (75.0)	348/625 (56.0)	0.086
Age (years) (median [IQR])	56 (45–35)	51 (33–57)	45 (32–59)	0.787†
> 6 years of formal schooling	432/645 (67.0)	9/20 (45.0)	423/625 (68.0)	0.034
House with dirt floor	154/645 (24.0)	3/20 (15.0)	151/625 (24.0)	0.344
Rural residence	81/624 (13.0)	3/18 (17.0)	78/606 (13.0)	0.637
Nearest health center distance (meters) (median [IQR])	689 (412–1095)	848 (274–1260)	692 (412–1092)	0.717†
Access to social security	225/645 (35.0)	3/20 (15.0)	222/625 (36.0)	0.058
Used alcohol	269/644 (42.0)	10/20 (50.0)	259/624 (42.0)	0.448
Used tobacco	139/644 (22.0)	8/20 (40.0)	131/624 (21.0)	0.042
Used illegal drugs	26/645 (4.0)	0/20 (0.0)	26/625 (4.0)	0.352
Homelessness or residence in shelters	14/644 (2.0)	0/20 (0.0)	14/624 (2.0)	0.498
Diabetes mellitus type 2	221/645 (34.0)	7/20 (35.0)	214/625 (34.0)	0.944
HIV coinfection	6/635 (1.0)	0/19 (0.0)	6/616 (1.0)	0.666
New case	592/644 (92.0)	17/20 (85.0)	575/624 (92.0)	0.248
AFB sputum positivity grade of 3 +	174/645 (27.0)	7/20 (35.0)	167/625 (27.0)	0.411
Resistance to at least one drug	93/522 (18.0)	13/17 (76.0)	80/505 (16.0)	< 0.001
MDR	11/522 (2.0)	5/17 (29.0)	6/505 (1.0)	< 0.001
Fever	462/642 (72.0)	11/20 (55.0)	451/622 (73.0)	0.086
Haemoptysis	225/645 (35.0)	6/20 (30.0)	219/625 (35.0)	0.642
Cavitations presence on chest radiograph	244/545 (45.0)	8/13 (62.0)	236/532 (44.0)	0.218
BMI (median [IQR])	21.0 (18.7–23.8)	19.1 (17.6–22.5)	21.1 (18.7–22.5)	0.181†
Number of days between symptom onset and start of treatment (median [IQR])	105 (67–197)	152 (79–242)	105 (66–179)	0.120†
*Lineage by MIRU*				
Haarlem	358/645 (55.5)	5/20 (25.0)	353/625 (56.5)	0.005‡
LAM	135/645 (20.9)	6/20 (30.0)	129/625 (20.6)	0.311‡
RD^Rio^	93/135 (68.9)	1/6 (16.7)	92/129 (71.3)	0.005‡
RD115	26/135 (19.3)	2/6 (33.3)	24/129 (18.6)	0.371‡
Other than Haarlem and LAM	152/645 (24.0)	9/20 (45.0)	143/625 (23.0)	0.022‡
Cluster belonging	447/645 (69.3)	10/20 (50)	437/625 (69.9)	0.057

IQR, interquartile range; HIV, human immunodeficiency virus; AFB, acid fast bacilli; MDR, multidrug resistance; BMI, body mass index; RD, region of difference

*Pearson chi-square test

^†^Kruskal–Wallis test

^‡^Binomial test

^a^Because there were missing values for the characteristics of some of the tuberculosis patients, several of the numbers below do not sum to the group total

We compared cavitation presence or absence with population clinical characteristics (Table 4). We found that patients with cavitation had DM2 as comorbidity (106/282, 38.0% vs 95/344, 28.0%; p = 0.008), presented greater number of AFB in sputum samples (101/282, 36.0% vs 71/344, 21.0%, p < 0.001) and presented LAM sublineage isolates (74/282, 26.2% vs 62/344, 18.0%; p = 0.013).

**Table 4:** Sociodemographic and clinical characteristics of patients with pulmonary tuberculosis by cavitation presentation, Orizaba Veracruz 1997–2010 (n = 755)

Variables	Total	Cavitations presence	Cavitations absence	*p-*value*
	n/total (%)	n/total (%)	n/total (%)	
Male	362/626 (58.0)	160/282 (57.0)	202/344 (59.0)	0.617
Age (years) (median [IQR])	46 (32–59)	47 (33–60)	45 (31–58	0.466†
> 6 years of formal schooling	429/626 (69.0)	191/282 (68.0)	238/344 (69.0)	0.696
House with dirt floor	151/626 (24.0)	66/282 (23.0)	85/344 (25.0)	0.704
Rural residence	81/610 (13.0)	36/273 (13.0)	45/337 (13.0)	0.952
Nearest health center distance (meters) (median [IQR])	702 (434.0–1089)	749 (464.1–1109)	683 (412–1073)	0.114†
Access to social security	224/626 (36.0)	108/282 (38.0)	116/344 (34.0)	0.235
Used alcohol	265/626 (42.0)	129/282 (46.0)	136/344 (40.0)	0.118
Used tobacco	135/626 (22.0)	54/282 (19.0)	81/344 (24.0)	0.183
Used illegal drugs	30/626 (5.0)	12/282 (4.0)	18/344 (5.0)	0.569
Homelessness or residence in shelters	18/626 (3.0)	10/282 (4.0)	8/344 (2.0)	0.363
Diabetes mellitus type 2	201/626 (32.0)	106/282 (38.0)	95/344 (28.0)	0.008
HIV coinfection	11/616 (2.0)	2/276 (1.0)	9/340 (3.0)	0.073
New case	566/625 (91.0)	249/281 (89.0)	317/344 (92.0)	0.132
AFB sputum positivity grade of 3+	172/626 (27.0)	101/282 (36.0)	71/344 (21.0)	< 0.001
Resistance to at least one drug	101/524 (19.0)	47/220 (21.0)	54/304 (18.0)	0.302
MDR	18/524 (3.0)	8/220 (4.0)	10/304 (3.0)	0.830
Fever	438/623 (70.0)	206/280 (74.0)	232/343 (68.0)	0.107
BMI (median [IQR])	21.0 (18.4–13.8)	20.8 (18.5–23.5)	21.2 (18.4–23.9	0.356†
Number of days between symptom onset and start of treatment (median [IQR])	104 (66–177)	108 (68–195)	100 (63–161)	0.064†
Haemoptysis	211/625 (34.0)	95/282 (34.0)	116/343 (34.0)	0.972
*Lineage by MIRU*				
Haarlem	344/626 (55.0)	152/282 (53.9)	192/344 (55.8)	0.632‡
LAM	136/626 (21.7)	74/282 (26.2)	62/344 (18.0)	0.013‡
RD^Rio^	97/136 (71.3)	55/74 (74.3)	42/62 (67.7)	0.398‡
RD115	24/136 (17.7)	11/74 (14.9)	13/62 (21.0)	0.352‡
Other than Haarlem and LAM	146/626 (23.0)	56/282 (20.0)	90/344 (26.0)	0.063‡‡
Cluster belonging	433/626 (69.2)	198/282 (70.2)	235/344 (68.3)	0.609‡

IQR, interquartile range; HIV, human immunodeficiency virus; AFB, acid fast bacilli; MDR, multidrug resistance; BMI, body mass index; RD, region of difference

*Pearson chi-square test

^†^Kruskal–Wallis test

^‡^Binomial test

Using logistic regression models, we performed two adjusted models to explain treatment failure compared to cure and treatment completion; in one we included resistance variable to at least one drug, and in the other we included MDR variable (Table 5). In the first model adjusted for covariates, treatment failure was associated with resistance to at least one drug (aOR 25.763, 95% CI 7.096–93.543; p < 0.001) and having lineage other than Haarlem and LAM (aOR 6.740, 95% CI 1.704–26.661; p = 0.007). In the model that included MDR variable adjusted for covariates, failure was associated with MDR (aOR 31.497, 95% CI 5.119–193.815; p < 0.001), in both models having > 6 years of formal education was not associated with treatment failure (aOR 0.166, 95% CI 0.045–0.615; p = 0.007), (aOR 0.248, 95% CI 0.069–0.885; p = 0.032) respectively.

**Table 5:** Characteristics associated with treatment failure Orizaba, Veracruz, 1997–2010 (n = 522)

Variables	aOR	95% CI		*p-*value*	aOR	95% CI		*p-*value*
		L.I	U.I			L.I	U.I	
Sex (male)	3.030	0.766	11.984	0.114	3.152	0.810	12.266	0.098
Age (years)	1.042	1.000	1.086	0.051	1.034	0.995	1.074	0.085
> 6 years of formal schooling	0.166	0.045	0.615	0.007	0.248	0.069	0.885	0.032
Access to social security	0.246	0.056	1.085	0.064	0.264	0.062	1.123	0.071
Diabetes mellitus type 2	0.716	0.195	2.634	0.615	0.750	0.222	2.532	0.643
New case	0.860	0.174	4.257	0.853	1.047	0.201	5.442	0.957
Resistance to at least one drug	25.763	7.096	93.543	< 0.001	–	–	–	–
MDR	–	–	–	–	31.497	5.119	193.815	< 0.001
*Lineage*								
Haarlem	Reference				Reference			
LAM	2.852	0.574	14.181	0.200	2.009	0.426	9.475	0.378
Other than Haarlem and LAM	6.740	1.704	26.661	0.007	3.342	0.906	12.320	0.070

aOR, adjusted odds ratio; MDR, multidrug resistance; L.I, lower interval; U.I, upper interval

*Adjusted logistic regression

We performed an adjusted logistic regression model to identify variables associated with cavitations presence (Table 6). Cavitation presence was associated with having ever consumed alcohol (aOR 1.528, 95% CI 1.041–2.243; p = 0.030), having DM2 (aOR 1.625, 95% CI 1.130–2.337; p = 0.009), AFB sputum positivity grade of 3 + (aOR 2.198, 95% CI 1.524–3.168; p < 0.001) and having the LAM sublineage (aOR 1.023, 95% CI 1.023–2.333; p = 0.039).

**Table 6:** Characteristics associated with the presence of cavitations in chest radiographs of patients with pulmonary TB in Orizaba, Veracruz, 1997–2010 (n = 626)

Variables	aOR	95% CI		*p-*value*
		L.I	U.I	
Sex (male)	0.781	0.529	1.152	0.212
Age (years)	1.003	0.993	1.013	0.573
Used alcohol	1.528	1.041	2.243	0.030
Diabetes mellitus type 2	1.625	1.130	2.337	0.009
AFB sputum positivity grade of 3+	2.198	1.524	3.168	< 0.001
*Lineage*				
Haarlem	Reference			
LAM	1.545	1.023	2.333	0.039
Other than Haarlem and LAM	0.806	0.537	1.210	0.298

aOR, adjusted odds ratio; MDR, multidrug resistance; L.I, lower interval; U.I, upper interval

*Adjusted logistic regression

## Discussion

This study describes the association among clinical and sociodemographic characteristics of patients with pulmonary tuberculosis and little described *M. tuberculosis* sublineages lineage 4 in the health jurisdiction of Orizaba, Veracruz, Mexico between 1995 and 2010. Our study population presented high frequency of lineage 4, Euro-American isolates. In addition, associated characteristics with treatment failure and cavitation presence were identified.

In this study, lineage 4 (Euro-American) was the most common (~ 96%) lineage identified, consistent with previous reports that have shown that isolates with this lineage are predominant in Mexico [18]. We were also able to observe that isolates with LAM lineage (163), the proportion of RD^Rio^ was 69.9%, higher compared to the 63.1% recently described in isolates from Northern Mexico and in isolates from Venezuela (55%), Argentine (11%) and Paraguay (10%) [19, 20]. Therefore, our results support that these lineages are endemic and that strains spread regionally with different rates of distribution.

We found that compared with other sublineages, cases with Haarlem sublineage isolates had higher proportion of clustered patients. A previous study showed similar results; the authors found that Haarlem sublineage isolates were more likely to belong to clusters [21]. This result confirms the wide distribution and genetic diversity of lineage 4 due to its virulence, which is reflected in cluster formation and its transmission success among the population [22].

On the other hand, we found that patients with Cameroon sublineage isolates showed more days between symptom onset and treatment start. Similar result have been described in patients with lineage 7 isolates in Ethiopia, where the time was longer between symptom onset and treatment start was attributed to lineage 7 strains slow growth [21]. Because treatment initiation is important to cut transmission chains, it is necessary to phenotypically confirm Cameroon sublineage isolates growth rate. To confirm this hypothesis, we cultured 45 isolates with Cameroon lineage on MGIT medium and determined the time and units of growth. We observed, that the Cameroon isolates grew less (14.6 CFU/h) compared to H37Rv (24.7 CFU/h).

Respect to Ghana sublineage, we found the majority of patients presented haemoptysis; this finding has not been reported thus far in literature. However, more data are needed.

Another interesting result was that cases with isolates of EAI lineage were more frequent in men, in patients with DM2 and patients who had ever smoked. It has been described that DM2 alone is associated with *M. tuberculosis* infection and progression to active disease with severe disease presentation [23]. Furthermore, decreased lung function has been observed in smokers with DM2 compared to nonsmokers [24]. Therefore, it is likely that social factors contribute to EAI dissemination, also these patients showed higher cavitations proportion (69.2%), without statistical significance. Previously, a study that evaluated host–pathogen relationship and its association with clinical outcomes in patients with tuberculosis described that patients infected with strains that originated in geographic regions other than the patient’s origin (allopatric) such as EAI lineage in America presented an increased lung damage risk [25]. As observed in our results, it has been suggested that although these lineages are less adapted to transmit and cause disease in fully competent members of allopatric human populations, they can do so in the context of impaired host immune resistance [26]. However, it would be necessary to perform whole genome sequence on EAI lineage isolates to determine pathogen genetic characteristics that facilitate its possible adaptation to the host and transmission.

Furthermore, East Asia (Beijing) lineage was found in two elderly patients, one of them had HIV and the other had DM2 and MDR. Beijing isolates were genetically distinct, with 9/15 different alleles by 24-loci MIRU–VNTR; these cases were probably due to reactivation. MDR has been associated with Beijing family; however, in this study, data are not conclusive because there were only two isolates [11, 27]. However, it is very likely that MDR isolate is due to antibiotics selective pressure because patient had received treatment previously.

We also observed higher proportion of treatment failure in patients with isolates of sublineages other than Haarlem and LAM, in patients who had ever smoked and in patients with isolates resistant to at least one drug or MDR. A greater proportion of resistance was found in Cameroon (13/49, 30.2%), UgandaI (5/22, 22.7%) and Ghana (2/16, 12.5%) sublineage isolates. A recent study conducted in Niger reported that 75% of Cameroon and Ghana sublineage isolates were resistant to RIF and MDR [28]. However, treatment failure could be also the result of "antibiotic resilience" as recently described by Quingyun et al., they found that mutations in *resR* (Rv1830) gene do not show canonical drug resistance or drug tolerance but instead shorten the post-antibiotic effect, meaning that they enable *M. tuberculosis* to resume growth after drug exposure substantially faster than wild-type strains, and these mutations are associated to treatment failure acting in a regulatory cascade with other transcription factors controlling cell growth and division. Furthermore, they described that up to 10% of strains from high-tuberculosis-burden countries showed fixed mutations in these regions [29].According to our results Cameroon and Ghana sublineages geographically restricted within Euro-American lineage, seem to have adapted to the study population and contribute significantly to the resistance generation and treatment failure. Therefore, it is necessary to genotype a greater number of isolates and performs susceptibility tests to determine the real impact on the resistance of lineages little described in Mexico and to perform whole genome sequencing to explore the possible association between *resR* mutation, treatment failure and whether any lineage is prone to acquire it.

Interestingly, having > 6 years of formal education was not associated with treatment failure. We believe that having higher education level probably implies that patients better understand treatment adherence and completion importance.

The LAM RD^Rio^ lineage has been described in other Latin American countries where it has been associated with the presence of cavitations, increased transmissibility and MDR [19]. However, in the present study we observed more proportion of LAM RD^Rio^ isolates in cured patients, previously in this study population was obtained that previous treatment (aOR 9.05, 95% CI 3.6–22.5, p < 0.001) and LAM lineage (aOR 4.25, 95% CI 1.4–12.7, p = 0.010) were associated with tuberculosis MDR [15]. These results have important implications in the tuberculosis control program, although isolates with LAM RD^Rio^ sub lineage are more prone to develop MDR following a previous treatment, patients seem to respond favorably to the second treatment.

Cavitations presence was associated with LAM sublineage, alcohol consumption, DM2 and AFB positivity grade 3+ . Similar results have been previously described regarding the presence of more severe manifestations in patients with DM2 and tuberculosis [30, 31]. Moreover, it has been reported that cavitations presence in pulmonary tuberculosis is associated with higher contagiousness/transmissibility due to high AFB load [32]. In addition, these results support those described by Pasopanodya et al., who report that modern lineages strains, such as Euro-American lineages, developed nonlethal properties; however, they cause lung damage, which increases their dissemination capacity among the population [25]. Therefore, the increase in the number of people with DM2 in Mexico could result in greater transmission of tuberculosis due to lung damage associated with the presence of LAM sublineage. We thus suggest implementing genotyping of *M. tuberculosis* isolates with the use of 24-loci MIRU–VNTR in Mexico and determining the impact of LAM sublineage.

In conclusion, this study provides relevant results in relation to the association between the presence of cavitations, comorbidities and LAM sublineage isolates. Additionally, treatment failure associated with sublineages other than Haarlem and LAM. Furthermore, we found the possible EAI sublineage isolates association in patients with DM2 and cavitation. We describe that the genetic diversity of *M. tuberculosis* lineage 4 (Euro-American) probably plays an essential role in disease presentation, which could have important implications for treatment management and to improve tuberculosis control in Mexico.

## Supplementary Information

**Additional file 1: Table S1. **Sociodemographic and clinical characteristics of patients with pulmonary tuberculosis according to disaggregated sublineage, determined by 24-*loci* MIRU-VNTR, Orizaba Veracruz 1997-2010 (n=755). **Table S2**. Treatment outcome of patients with pulmonary tuberculosis according to disaggregated lineage by 24-*loci *MIRU-VNTR, Orizaba Veracruz 1997-2010 (n=755). **Table S3.** Treatment outcome of patients with pulmonary tuberculosis according to aggregated lineage by 24-*loci *MIRU-VNTR, Orizaba Veracruz 1997-2010 (n=755).

## Data Availability

The datasets used during the current study are available from the corresponding author upon reasonable request.

## Abbreviations

aOR: Adjusted odds ratio
AFB: Acid-fast bacilli
BMI: Body mass index
CIs: Confidence intervals
DM2: Diabetes mellitus type 2
EMB: Ethambutol
HIV: Human immunodeficiency virus
INH: Isoniazide
IQR: Interquartile ranges
LAM: Latin American & Mediterranean
MDR-TB: Tuberculosis multidrug-resistant
MIRU–VNTR: Variable number tandem repeat–mycobaterial interspersed repetitive unit
MTBC: *Mycobacterium tuberculosis* complex
RIF: Rifampicin
STR: Streptomycin
