# Supplementary material for: Mycobacterium tuberculosis lineage 4 associated with cavitations and treatment failure
Source: BMC Infect Dis. 2023 Mar 14;23:154. doi: 10.1186/s12879-023-08055-9 (PMC10012486; doi:10.1186/s12879-023-08055-9)
Supplement: Supplementary file 1 — Additional file 1: Table S1. Sociodemographic and clinical characteristics of patients with pulmonary tuberculosis according to disaggregated sublineage, determined by 24-loci MIRU-VNTR, Orizaba Veracruz 1997-2010 (n=755). Table S2. Treatment outcome of patients with pulmonary tuberculosis according to disaggregated lineage by 24-loci MIRU-VNTR, Orizaba Veracruz 1997-2010 (n=755). Table S3. Treatment outcome of patients with pulmonary tuberculosis according to aggregated lineage by 24-loci MIRU-VNTR, Orizaba Veracruz 1997-2010 (n=755). [file 12879_2023_8055_MOESM1_ESM.docx]

**Additional file 1**

**Table S1.** Sociodemographic and clinical characteristics of patients with pulmonary tuberculosis according to disaggregated sublineage, determined by 24-*loci* MIRU-VNTR, Orizaba Veracruz 1997-2010 (n=755)

| **Variables** | **Total** | **Haarlem** | **LAM** | **Cameroon** | **UgandaI** | **Ghana** | **S** | **X** | **TUR** | **EAI** | **Beijing** | **Unknown** | ***p-*value*** |
| --- | --- | --- | --- | --- | --- | --- | --- | --- | --- | --- | --- | --- | --- |
|  | **n/total (%)** | **n/total (%)** | **n/total (%)** | **n/total (%)** | **n/total (%)** | **n/total (%)** | **n/total (%)** | **n/total (%)** | **n/total (%)** | **n/total (%)** | **n/total (%)** | **n/total (%)** |  |
| Male sex | 442/755 (58.5) | 257/419 (61.3) | 81/163 (49.7) | 30/49 (61.2) | 21/28 (75.0) | 9/23 (39.1) | 12/18 (66.7) | 7/15 (46.7) | 8/15 (53.3) | 12/15 (80.0) | 0/2 (0.00) | 5/8 (62.5) | 0.023 |
| Age (years) (median [IQR]) | 45 (32-59) | 45 (33-59) | 43 (30-55) | 38 (24-53) | 45 (29-58) | 58 (34-70) | 46 (38-59) | 56 (32-77) | 38 (29-54) | 53 (41-64) | 59 (57-60) | 45 (27-62) | 0.040 |
| >6 years of formal schooling | 507/754 (67.2) | 277/418 (66.3) | 115/163 (70.6) | 26/49 (53.1) | 20/28 (71.4) | 19/23 (82.6) | 13/18 (72.2) | 12/15 (80.0) | 9/15 (60.0) | 8/15 (53.3) | 2/2 (100) | 6/8 (75.0) | 0.264 |
| House with dirt floor | 174/755 (23.1) | 88/419 (21.0) | 44/163 (27.0) | 9/49 (18.4) | 9/28 (32.1) | 6/419 (21.0) | 5/18 (27.8) | 4/15 (26.7) | 4/15 (26.7) | 2/15 (13.3) | 0/2 (0.0) | 3/8 (37.5) | 0.703 |
| Rural residence | 99/732 (13.5) | 47/409 (11.5) | 30/159 (18.9) | 3/47 (6.4) | 5/26 (19.2) | 3/21 (14.3) | 2/18 (11.1) | 4/15 (26.7) | 2/14 (14.3) | 1/15 (6.7) | 0/1 (0.0) | 2/7 (28.6) | 0.251 |
| Nearest health center distance (meters) (median [IQR]) | 708 (414-1,099) | 685 (404-1025) | 775 (432-1184) | 735 (476-1044) | 760 (324-1449) | 674 (267-1130) | 566 (471-861) | 899 (519-1413) | 808 (484-1432) | 1045 (609-1109) | 388 (354-422) | 1504 (853-2094) | 0.154 |
| Access to social security | 254/755 (33.6) | 152/419 (36.3) | 48/163 (29.5) | 12/49 (24.5) | 10/28 (35.7) | 9/23 (39.1 | 4/18 (22.2) | 5/15 (33.3) | 5/15 (33.3) | 7/15 (46.7) | 1/2 (50.0) | 1/8 (12.5) | 0.549 |
| Used alcohol | 330/753 (43.8) | 194/417 (46.5) | 64/163 (39.3) | 22/49 (44.9) | 17/28 (60.7) | 7/23 (30.4) | 8/18 (44.4) | 2/15 (33.3) | 6/15 (40.0) | 8/15 (53.3) | 0/2 (0.0) | 2/8 (25.0) | 0.084 |
| Used tobacco | 164/753 (21.8) | 106/417 (25.4) | 22/163 (13.5) | 10/49 (20.4) | 5/28 (17.9) | 2/23 (8.70) | 6/18 (33.3) | 1/15 (6.7) | 5/15 (33.3) | 7/15 (46.7) | 0/2 (0.0) | 2/8 (0.0) | 0.005 |
| Used illegal drugs | 35/754 (4.6) | 19/418 (4.6) | 5/163 (3.1) | 3/49 (6.1) | 2/28 (7.1) | 0/23 (0.0) | 2/18 (11.1) | 0/15 (0.0) | 2/15 (13.3) | 2/15 (13.3) | 0/2 (0.0) | 2/8 (0.0) | 0.380 |
| Homelessness or residence in shelters | 20/754 (2.7) | 12/419 (2.9) | 4/162 (2.5) | 0/49 (0.0) | 1/28 (3.6) | 1/23 (4.4) | 0/18 (0.0) | 0/15 (0.0) | 1/15 (6.7) | 1/15 (6.7) | 0/2 (0.0) | 0/8 (0.0) | 0.903 |
| Diabetes mellitus type 2 | 250/755 (33.1) | 138/419 (32.9) | 52/163 (31.9) | 12/49 (24.5) | 11/28 (39.3) | 7/23 (30.4) | 6/18 (33.3) | 6/15 (40.0) | 6/15 (40.0) | 10/15 (66.7) | 1/2 (50.0) | 1/8 (12.5) | 0.261 |
| HIV coinfection | 13/739 (1.8) | 7/410 (1.7) | 3/163 (1.8) | 1/47 (2.1) | 1/27 (3.7) | 0/23 (0.0) | 0/16 (0.0) | 0/14 (0.0) | 0/14 (0.0) | 0/15 (0.0) | 1/2 (50.0) | 0/8 (0.0) | 0.001 |
| New case | 688/754 (91.3) | 384/418 (91.9) | 145/163 (89.0) | 47/49 (95.9) | 26/28 (92.9) | 21/23 (91.3) | 18/18 (100.0) | 12/15 (80.0) | 14/15 (93.3) | 14/15 (93.3) | 1/2 (50.0) | 6/8 (75.0) | 0.179 |
| AFB sputum positivity grade of 3+ | 211/755 (28.0) | 123/419 (29.4) | 47/163 (28.8) | 13/49 (26.5) | 10/28 (35.7) | 3/23 (13.0) | 5/18 (27.8) | 4/15 (26.7) | 1/15 (6.7) | 4/15 (26.7) | 0/2 (0.0) | 1/8 (12.5) | 0.530 |
| Resistance to at least one drug | 117/612 (19.1) | 68/337 (20.2) | 25/131 (19.1) | 13/43 (30.2) | 5/22 (22.7) | 2/16 (12.5) | 0/14 (0.0) | 0/14 (0.0) | 0/12 (0.0) | 2/14 (14.3) | 1/2 (50.0) | 1/7 (14.3) | 0.121 |
| MDR | 21/612 (3.4) | 8/337 (2.4) | 8/131 (6.1) | 2/43 (4.7) | 1/22 (4.55) | 0/16 (0.0) | 0/14 (0.0) | 0/14 (0.0) | 0/12 (0.0) | 0/14 (0.0) | 1/2 (50.0) | 1/7 (14.3) | 0.014 |
| Fever | 531/752 (70.6) | 292/417 (70.0) | 114/162 (70.4) | 36/49 (73.5) | 23/28 (82.1) | 14/23 (60.99 | 11/18 (61.1) | 13/15 (86.7) | 12/15 (80.0) | 11/15 (73.3) | 1/2 (50.0) | 4/8 (50.0) | 0.580 |
| Haemoptysis | 250/753 (33.2) | 141/417 (33.8) | 39/163 (23.9) | 19/49 (38.8) | 13/28 (46.4) | 11/23 (47.8) | 8/18 (44.4) | 7/15 (46.7) | 5/15 (33.3) | 7/15 (46.7) | 0/2 (0.0) | 0/8 (0.0) | 0.030 |
| Cavitations presence on chest radiograph | 282/626 (45.1) | 152/344 (44.2) | 74/136 (54.5) | 11/40 (27.5) | 12/24 (50.0) | 8/19 (42.1) | 7/17 (41.2) | 3/12 (25.0) | 3/12 (25.0) | 9/13 (69.2) | 1/2 (50.0) | 2/7 (28.6) | 0.054 |
| BMI (median [IQR]) | 20.9 (18.4-23.8) | 20.5 (18.1-23.4) | 21.0 (18.9-24.1) | 20.3 (18.9-22.4) | 21.6 (18.7-24.1) | 22.7 (19.0-24.2) | 20.9 (18.0-24.1) | 23.0 (19.1-24.9) | 23.9 (22.2-25.7) | 22.1 (18.7-24.0) | 25.4 (21.6-29.3) | 21.3 (18.6-22.6) | 0.053 |
| Number of days between symptom onset and start of treatment (median [IQR]) | 105  (67-182) | 106  (63-180) | 119 (80-196) | 129  (83-198) | 122  (88-284) | 85 (28-131) | 102 (66-215) | 82 (53-155) | 68 (51-96) | 76 (48-92) | 101 (22-179) | 112  (82-136) | 0.017 |
| Cluster belonging | 519/755 (68.7) | 318/419 (75.9) | 95/163 (58.3) | 36/49 (73.5) | 17/28 (60.7) | 12/23 (52.2) | 8/18 (44.4) | 9/15 (60.0) | 11/15 (73.3) | 10/15 (66.7) | 0/2 (0.0) | 3/8  (37.5) | <0.001 |

Abbreviations: IQR-interquartile range; HIV-human immunodeficiency virus; AFB-acid fast bacilli; MDR-multidrug resistance; BMI-body mass index.

* Pearson chi-square test

† Kruskal Wallis test

**Table S2**. Treatment outcome of patients with pulmonary tuberculosis according to disaggregated lineage by 24-*loci* MIRU-VNTR, Orizaba Veracruz 1997-2010 (n=755)

| **Variables** | **Total** | **Haarlem** | **LAM** | **Cameroon** | **UgandaI** | **Ghana** | **S** | **X** | **TUR** | **EAI** | **Beijing** | **Unknown** | ***p-*value*** |
| --- | --- | --- | --- | --- | --- | --- | --- | --- | --- | --- | --- | --- | --- |
|  | **n/total**  **(%)** | **n/total**  **(%)** | **n/total (%)** | **n/total**  **(%)** | **n/total (%)** | **n/total (%)** | **n/total (%)** | **n/total (%)** | **n/total (%)** | **n/total (%)** | **n/total (%)** | **n/total**  **(%)** |  |
| **Treatment outcome** | | | | | | | | | | | | |  |
| Abandon | 49/755  (6.5) | 27/419  (6.4) | 13/163  (8.0) | 3/49  (6.1) | 0/28  (0.0) | 0/23  (0.0) | 0/18  (0.0) | 4/15  (26.7) | 1/15  (6.7) | 0/15  (0.0) | 0/2  (0.0) | 1/8  (12.5) | 0.059 |
| Cure | 532/755 (70.5) | 306/419 (73.0) | 109/163 (66.9) | 28/49  (57.1) | 23/28 (82.1) | 15/23  (65.2) | 14/18 (77.8) | 8/15  (53.3) | 9/15  (60.0) | 13/15  (86.7) | 2/2  (100) | 5/8  (62.5) |  |
| Treatment completion | 93/755  (12.3) | 47/419  (11.2) | 20/163  (12.3) | 10/49  (20.4) | 2/28  (7.1) | 6/23  (26.1) | 3/18  (16.7) | 1/15  (6.7) | 2/15  (3.3) | 1/15  (6.7) | 0/2  (0.0) | 1/8  (12.5) |  |
| Failure | 20/755  (2.7) | 5/419  (1.2) | 6/163  (3.7) | 3/49  (6.1) | 1/28  (3.6) | 0/23  (0.0) | 1/18  (5.6) | 2/15  (13.3) | 0/15  (0.0) | 1/15  (6.7) | 0/2  (0.0) | 1/8  (12.5) |  |
| Death during treatment | 26/755  (3.4) | 19/419  (4.5) | 4/163  (2.5) | 2/49  (4.1) | 1/28  (3.6) | 0/23  (0.0) | 0/18  (0.0) | 0/15  (0.0) | 0/15  (0.0) | 0/15  (0.0) | 0/2  (0.0) | 0/8  (0.0) |  |
| No data | 35/755  (4.6) | 15/419  (3.6) | 11/163  (6.8) | 3/49  (6.1) | 1/28  (3.6) | 2/23  (8.7) | 0/18  (0.0) | 0/15  (0.0) | 3/15  (20.0) | 0/15  (0.0) | 0/2  (0.0) | 0/8  (0.0) |  |
| **Treatment failure** | | | | | | | | | | | | |  |
| Failure | 20/645  (3.1) | 5/358  (1.4) | 6/135  (4.4) | 3/41  (7.3) | 1/26  (3.9) | 0/21  (0.0) | 1/18  (5.6) | **2/11**  **(18.2)** | 0/11  (0.0) | 1/15  (6.7) | 0/2  (0.0) | **1/7**  **(14.3)** | 0.029 |
| Cure or treatment completion | 625/645 (96.9) | 353/358 (98.6) | 129/135 (95.6) | 38/41  (92.7) | 25/26 (96.2) | 21/21  (100) | 17/18 (94.4) | 9/11  (81.2) | 11/11  (100) | 14/15 (93.3) | 2/2  (100) | 6/7  (85.7) |  |
| **No treatment success** | | | | | | | | | | | | |  |
| Abandon, failure or death during treatment | 95/720  (13.2) | 51/404  (12.6) | 23/152  (15.1) | 8/46  (17.4) | 2/27  (7.4) | 0/21  (0.0) | 1/18  (85.6) | 6/15  (40.0) | 1/12  (8.3) | 1/15  (6.7) | 0/2  (0.0) | 2/8  (25.0) | 0.060 |
| Cure or treatment completion | 625/720 (86.8) | 353/404 (87.4) | 129/152 (84.9) | 38/46  (82.6) | 25/27 (92.6) | 21/21  (100) | 17/18 (94.4) | 9/15  (60.0) | 11/12 (91.7) | 14/15 (93.3) | 2/2  (100) | 6/8  (75.0) |  |
| **Death during treatment** | | | | | | | | | | | | |  |
| Death | 26/720  (3.6) | 19/404  (4.7) | 4/152  (2.6) | 2/46  (4.4) | 1/27  (3.7) | 0/21  (0.0) | 0/18  (0.0) | 0/15  (0.0) | 0/12  (0.0) | 0/15  (0.0) | 0/2  (0.0) | 0/8  (0.0) | 0.871 |
| Cure or treatment completion, abandon or failure | 694/720 (96.4) | 385/404 (95.3) | 148/152 (97.4) | 44/46  (95.7) | 26/27 (96.3) | 21/21  (100) | 18/18  (100) | 15/15  (100) | 12/12  (100) | 15/15  (100) | 2/2  (100) | 8/8  (100) |  |

* Pearson chi-square test

**Table S3.** Treatment outcome of patients with pulmonary tuberculosis according to aggregated lineage by 24-*loci* MIRU-VNTR, Orizaba Veracruz 1997-2010 (n=755)

| **Variables** | **Total** | **Haarlem** | **LAM** | **Other than Haarlem and LAM** | ***p-*value*** |
| --- | --- | --- | --- | --- | --- |
|  | **n/total (%)** | **n/total (%)** | **n/total (%)** | **n/total (%)** |  |
| **Treatment outcome** |  |  |  |  |  |
| Abandon | 49/755 (6.5) | 27/419 (6.4) | 13/163 (8.0) | 9/173 (5.2) | 0.061 |
| Cure | 532/755 (70.5) | 306/419 (73.0) | 109/163 (66.9) | 117/173 (67.6) |  |
| Treatment completion | 93/755 (12.3) | 47/419 (11.2) | 20/163 (12.3) | 26/173 (15.0) |  |
| Failure | 20/755 (2.7) | 5/419 (1.2) | 6/163 (3.7) | 9/173 (5.2) |  |
| Death during treatment | 26/755 (3.4) | 19/419 (4.5) | 4/163 (2.5) | 3/173 (1.7) |  |
| No data | 35/755 (4.6) | 15/419 (3.6) | 11/163 (6.8) | 9/173 (5.2) |  |
| **Treatment failure** |  |  |  |  |  |
| Failure | 20/645 (3.1) | 5/358 (1.4) | 6/135 (4.4) | 9/152 (5.9) | 0.016 |
| Cure or treatment completion | 625/645 (96.9) | 353/358 (98.6) | 129/135 (95.6) | 143/152 (94.1) |  |
| **No treatment success** |  |  |  |  |  |
| Abandon, failure or death during treatment | 95/720 (13.2) | 51/404 (12.6) | 23/152 (15.1) | 21/164 (12.8) | 0.728 |
| Cure or treatment completion | 625/720 (86.8) | 353/404 (87.4) | 129/152 (84.9) | 143/164 (87.2) |  |
| **Death during treatment** |  |  |  |  |  |
| Death | 26/720 (3.6) | 19/404 (4.7) | 4/152 (2.6) | 3/164 (1.8) | 0.192 |
| Cure or treatment completion, abandon or failure | 694/720 (96.4) | 385/404 (95.3) | 148/152 (97.4) | 161/164 (98.2) |  |

* Pearson chi-square test
